# Hospital Meetings, &C.

**Published:** 1898-05-14

**Authors:** 


### HOSPITAL MEETINGS, &C.

### THE LORD MAYOR'S DINNER TO THE MEDICAL PROFESSION.

Speech by Lord Lansdowne on the Army Medical Service.

The following report of Lord Lansdowne's speech is taken from the *Times* of the 5th inst. :—

"Lord Lansdowne, in responding for the Army, said,—I am sure that the generous words of the Lord Chief Justice will be valued by all those who serve Her Majesty in the Army, and by all the friends of the Army. In this country we are notoriously apt to disparage our own institutions and to dwell more upon their faults than upon their merits, and I think it is true that this tendency is frequently exhibited when we discuss the British Army. Its recent performance shows, at any rate, that, whatever its imperfections, it contains soldiers who are able to uphold its great traditions under the most trying circumstances. The recent operations on the North-West Frontier of India and in Egypt have both illustrated in a conspicuous manner the bravery and resource of our troops. I am sure we must all have read with pleasure the dignified and soldierlike words, reported in this morning's papers, in which Sir William Lockhart took leave of the force which he lately commanded during the Tirah Expedition. The Frontier campaign and that now in progress in Egypt have this in common—that in both our soldiers have been fighting under the same flag with men not of our own race, but owing their value as soldiers to the training and discipline which they have received at our hands. (Cheers.) While we have in the British army such leaders as Sir William Lockhart and Sir Herbert Kitchener we need never despair. (Renewed cheers.) Military operations like these are of value to us in that we learn from them not only where we are strong, but where we are weak. The Egyptian campaign has apparently brought to light one weak point which we cannot afford to ignore. I refer to the boots with which we supplied our soldiers. Not that I have any doubt that the Army boot is a good boot—I believe it, on the contrary, to be an honestly-made and serviceable article—but it is apparently unsuited to resist the peculiar and insidious action of the desert sand. In saying a word for the boots, I am glad to be given an opportunity of saying a word for the wearers. The boots worn by the British Brigade in Egypt did not have a very easy time of it. I find that the British Brigade, led by my old friend, General Gatacre, when marching to the front covered a distance of no less than 142 miles in five days, with only one day's rest intervening. (Cheers.) That gives an average of 28½ miles a day, which, in the climate of the Soudan, I think you will agree is a memorable performance, all the more so because scarcely a man fell out. (Cheers.) I trust that we shall be able to invent a boot which even General Gatacre and the desert sand will not be able to wear out for us. (Cheers.) There is no department in the public service which owes more to, and depends more upon the members of your profession than the Army. In time of peace it is you who teach us how to maintain the health of our troops and to combat the diseases which they have to encounter in the various and often trying climates which prevail in different parts of our ever-increasing Empire. In time of war you do something to correct the barbarity of our methods and to mitigate the nameless sufferings which follow in the wake of great battles. With every year that passes the engines of destruction are brought to a greater pitch of perfection. The wounds which could now be inflicted in a few seconds are more numerous and more serious than could have been inflicted in the same number of hours not many years ago. Your skill and devotion are the correctives of our modern ruthlessness, and I think most of us will say that, if we had to choose between the credit belonging to the artillerist who has, let us say, invented a new form of dum-dum bullet and the credit belonging to the surgeon who has contrived the means of extracting it painlessly and saving the shattered limbs, we should not hesitate in deciding whose part we should prefer. (Cheers.) I am using no idle phrase when I say that the Army is proud that it contains a number of officers who belong to the medical profession, but who are none the less soldiers in the fullest sense of the word—(loud cheers)—wearing the Queen's uniform, holding her commission, ready to take their share, aye and more than their share, of the risk and hardship of warfare. (Cheers.) We are determined that there shall be no failure, either in theory or in practice, to treat them with the respect to which they are entitled. (Cheers.) I insist upon this point, because I have observed with very keen regret that there has existed for some time past a certain estrangement between the Army and the profession. This want of confidence has had for its result that we have been unable to attract to the service the number of candidates, and I am afraid I must add the class of candidates, whom we should like to see serving in the Medical Staff. I will not attempt to diagnose the case. Some of the symptoms are puzzling and obscure. It is possible that in this case, as in many others in which reasonable people have agreed to differ, mistakes have been made on both sides. We on our side are determined that you shall have no cause of complaint of us. (Cheers.) We have already shown by our action in dealing with several points to which you have called our attention that we were prepared to redress any injustice where you were able to satisfy us that injustice had been done. We are now about to deal, and to deal thoroughly, with a question in regard to which not only the Medical Staff of the Army, but the medical profession generally, feels most strongly—I mean the rank and status of the Army Medical Staff. I sometimes hear it said, 'What does rank matter? Is not the title of "Doctor" or "Surgeon" by itself to be regarded as a title which any one should be proud to wear without further adjuncts?' I think the answer to that is that in the Army rank is the outward and visible sign of consideration and authority, and that although 'a man may be a man for all that,' it is necessary, if he adopts the military profession, that he should have a proper military stamp to distinguish him and to secure him his proper place among his comrades. (Cheers.) We have attempted several times to deal with this question of rank as it affects the Army doctors, and we have not been particularly successful; we have invented ingenious compromises which satisfied nobody, and titles some of which would for cumbrousness and cacophony be hard to beat. We propose to make a fresh start and to form—out of the Army Medical Staff and the Medical Staff Corps—a corps the officers of which will bear the same miltary titles as other officers of the Army—(cheers)—always, of course, upon the clear understanding that those titles do not confer upon them any right of command outside it. We propose to give them these titles up to the rank of colonel. I have been pressed not to stop there, but to go on to the rank of general officer—(cheers)—but there are reasons which seem to me conclusive against doing this. Our present policy is to restrict as closely as we can the number of officers bearing the title of 'General,' and to insist that no one shall rise to that rank unless he is required to fill one of a limited number of appointments, all of which will involve general command in the Army and fitness, on occasion arising, to command troops in the field. No departmental officers will under this system be able to reach the rank of general officer, and it will be impossible for us to confer it upon members of the medical staff. Above the rank of colonel we propose to retain that of surgeon-general, with the rank and precedence of a general officer in the Army. (Loud cheers.) I have one thing to add. Her Majesty—upon whose goodwill towards your profession I need not dwell—has been pleased to signify her intention of bestowing upon the newly-formed corps the title of the Royal Army Medical Corps, a title which I am sure it will wear worthily. (Loud and prolonged cheers.) I trust that the members of the profession will recognise the sincerity with which we have endeavoured to meet them, and that they on their side will help us, to the utmost of their ability, to secure the services of the best of their students. (Cheers.) I am sure they desire as keenly as we do that wherever the British Army shall be found, whether in peace or war, there shall be found serving on its medical staff men who from their ability and character, and from their standing in their profession, shall be in every sense worthy representatives of a great and noble profession. (Loud cheers.)"

### ROYAL LONDON OPHTHALMIC HOSPITAL.

The first festival dinner held by the Royal London Ophthalmic Hospital since the early part of this century took place at the Grand Hotel, Northumberland Avenue, on Friday, the 6th inst. H.R.H. the Duke of Cambridge presided, and amongst those present were Sir John Lubbock, Bart., M.P., General Bateson, C.V.O., Sir Whittaker Ellis, Bart., Messrs. H. P. Sturgis (chairman of the committee) and Horace G. Bowen. The Chairman, in proposing "The Queen," said that Her Majesty was a patron of this hospital even before she came to the throne. In proposing "Prosperity to the Royal London Ophthalmic Hospital," the Chairman said that as the possibility of living depended almost entirely on the possession of sight, the necessity for such a hospital was undoubted. Referring to the removal of the institution to the new site, he said that it was hoped that the new building would be in occupation this year. This change in site would prove very expensive, and so it was necessary this year for all to be as liberal as possible in order that the new hospital should be as successful as one could wish it to be. Under the new arrangement there would be 140 beds instead of only 100, and many more cases could therefore be attended. He trusted that the gifts to the hospital would be as liberal as possible, for there could not be two opinions as to the advantage and the utility of the hospital. It was under excellent management, and there was every prospect of its extending its work in the future. (Applause.) Sir John Lubbock, Bart., responding, said that the hospital was the oldest ophthalmic hospital, not only in Great Britain but in the whole world. This was rather surprising, seeing that it only dated back to 1804. There was no charity which could appeal with more confidence to the generosity of the public than this hospital, because the saving to the community of which it was the cause was enormous. Take the case of cataract alone. About 500 cases were dealt with by the hospital in the year. If they reckoned each case as saving £250, that would represent a gain to the community of £100,000 a year. Of glaucoma the same might be said, and these were only two of the diseases which were dealt with at this hospital. They, therefore, had a very strong case on which to base an appeal for support. (Applause.) The Secretary announced that £1,435 had been collected as a result of the dinner. "The Medical Officers" was proposed by Mr. Horace G. Bowen, and responded to by Mr. John Tweedy. Other toasts followed.

### BRITISH HOME FOR INCURABLES.

The Earl Amherst, who presided at the annual general meeting held at the Cannon-street Hotel on Tuesday, had occasion to congratulate the governors and subscribers on the financial result of the year. The annual subscriptions amounted to £3,111, an increase on the previous year, the donations to £6,470, and the legacies to £4,805, the total income reaching the sum of £16,779, as compared with £14,034 for 1886-7. The surplus of income over expenditure was £4,063, which has been carried to the funds of the charity. The investments now stand at £54,095. In the past 12 months the sum paid to annuitants amounted to £6,159, the largest payment made by the charity in a single year. Dealing with the very satisfactory financial position, the report remarks that this has been achieved without any special Jubilee appeal. Attention is also directed the fact that for the first time in the history of the charity the beds have been fully occupied. At March 31st last there were 70 in-patients and 302 pensioners on the books, 14 in-patients having been admitted and seven having died during the year. The board announce the completion of the home by the alteration and enlargement of several rooms on the top floor, and state that the one thing now wanted is an entertainment hall for the recreation and amusement of the inmates. As foreshadowed last year, the seaside home at Margate has been closed, and the balance carried to a "Samaritan Fund." The adoption of the report having been seconded by Mr. J. H. Hale and carried, the election of inmates and annuitants was proceeded with.

